# Regional Peripheral Neuromodulation via Glucopuncture: A Novel Targeted Approach for Persistent Myofascial Dysfunction

**DOI:** 10.7759/cureus.93637

**Published:** 2025-10-01

**Authors:** King Hei Stanley Lam, Jan Kersschot, Teinny Suryadi, Anwar Suhaimi, Daniel Chiung-Jui Su

**Affiliations:** 1 Faculty of Medicine, The University of Hong Kong, Hong Kong, HKG; 2 Department of Clinical Research, The Hong Kong Institute of Musculoskeletal Medicine, Kowloon, HKG; 3 Department of Clinical Research, International Association of Musculoskeletal Medicine, Kowloon, HKG; 4 Department of Family Medicine, Private Practice, Antwerp, BEL; 5 Department of Physical Medicine and Rehabilitation, Synergy Clinic, Jakarta, IDN; 6 Department of Physical Medicine and Rehabilitation, Hermina Podomoro Hospital, Jakarta, IDN; 7 Department of Physical Medicine and Rehabilitation, Medistra Hospital, Jakarta, IDN; 8 Department of Rehabilitation Medicine, University Malaya Medical Centre, Kuala Lumpur, MYS; 9 Department of Rehabilitation Medicine, University Malaya, Kuala Lumpur, MYS; 10 Department of Physical Medicine and Rehabilitation, Chi Mei Medical Center, Tainan, TWN; 11 Department of Clinical Research, A Tempo Regeneration Center for Musicians, Tainan, TWN

**Keywords:** 5% dextrose in water without local anesthetic, biotensegrity, fascia system, glucopuncture, musculoskeletal pain, myofascial pain, pain management, perineural injection therapy, peripheral nerve dysfunction, prolotherapy

## Abstract

Chronic musculoskeletal pain syndromes with discordant clinical-radiological findings present significant therapeutic challenges. This study evaluates glucopuncture-a minimally invasive intervention targeting fascial and muscular pain generators-in two refractory cases: an elderly female patient with severe thumb osteoarthritis (Eaton-Littler stage III) and a professional kickboxer with persistent cervicobrachial pain. Both received standardized palpation-guided glucopuncture using 27-gauge needles (subcutaneous: 0.5 mL/site for fascial pain; intramuscular: 1 mL/site for myofascial dysfunction).

The geriatric patient achieved 90% pain reduction (Numerical Rating Scale (NRS) 8-9 to 0-1) and functional improvement (Quick Disabilities of the Arm, Shoulder, and Hand (QuickDASH) 55 to 25) after three sessions, sustained at three-month and one-year follow-ups. The athlete attained near-complete symptom resolution (QuickDASH 56.8 to 11.3; Neck Disability Index (NDI) 77 to 33) after five sessions, enabling full return to training with efficacy persisting at one year. Outcomes exceeded established minimal clinically important difference thresholds. Compared to alternatives, glucopuncture avoids intentional tissue injury (prolotherapy), supplements dry needling with biochemical modulation, and offers cost-effective simplicity versus platelet-rich plasma. Safety was favorable, with transient pain flares in <20% of interventions.

These preliminary findings support glucopuncture as a promising therapy for complex pain syndromes unresponsive to conventional treatments. Further randomized trials should evaluate dose optimization, long-term efficacy, and health economic impact.

## Introduction

Chronic musculoskeletal pain syndromes characterized by discordant clinical-radiological findings present significant therapeutic challenges in contemporary medicine. Standard management, often guided by imaging, regularly fails to address functional pain originating from fascial and muscular networks. This creates a substantial burden for patients and clinicians alike. Glucopuncture has emerged as a potential intervention for such stubborn conditions. This technique involves multiple injections of 5% dextrose (D5W) to modulate pain through proposed biomechanical and neuropharmacological pathways. This technique is theorized to target two interconnected physiological systems: the fascial system-a dynamic sensory and biomechanical matrix [1-3]-and the muscular system, which serves dual roles in movement execution and nociception.

The fascial system constitutes a vital yet historically understudied anatomical framework [4-7]. It is a continuous web of connective tissue that integrates the body's structures, including nerves, vessels, and muscles [1,5,6,8,9]. It also functions as a richly innervated sensory organ [10]. Recent research highlights its adaptive properties, sometimes termed "fascintegrity" [11], which may explain the pain radiation patterns and referral phenomena seen in fascial pain syndromes [5,12,13]. Critically, these functional disturbances frequently evade detection by conventional imaging modalities (radiographs, MRI [14], and ultrasound) [15], creating diagnostic dilemmas that contribute to therapeutic failures.

Similarly, the muscular system is a major source of pain. Minor, repetitive muscle injuries-common in overuse-can cause persistent pain and stiffness that does not respond well to standard therapies. Glucopuncture is proposed to act on both systems through several mechanisms. These include the downregulation of pain mediators [16-19], suppression of neuroinflammation [20], and the biomechanical normalization of fascial tension [21]. These actions are hypothesized to synergize with historical trigger point techniques [22] to target pain at its source rather than through symptom suppression.

Despite growing awareness of fascial and muscular pain, a significant gap remains in the availability of standardized, reproducible treatments for patients with normal imaging results. Previous studies have often lacked detailed methodology or a unified theory of action. Therefore, the primary objective of this manuscript is to present a standardized protocol for palpation-guided glucopuncture and report preliminary outcomes using objective measures (Numerical Rating Scale (NRS), Quick Disabilities of the Arm, Shoulder, and Hand (QuickDASH), and Neck Disability Index (NDI)). We also aim to unify the neuropharmacological and biomechanical theories into a coherent treatment model. By describing a methodology for managing fascial-muscular pain that is independent of structural imaging findings, this work provides a novel framework for clinicians and researchers investigating these complex conditions.

## Case presentation

Case 1: chronic fascial-mediated pain in geriatric basal joint osteoarthritis

**Figure 1:**
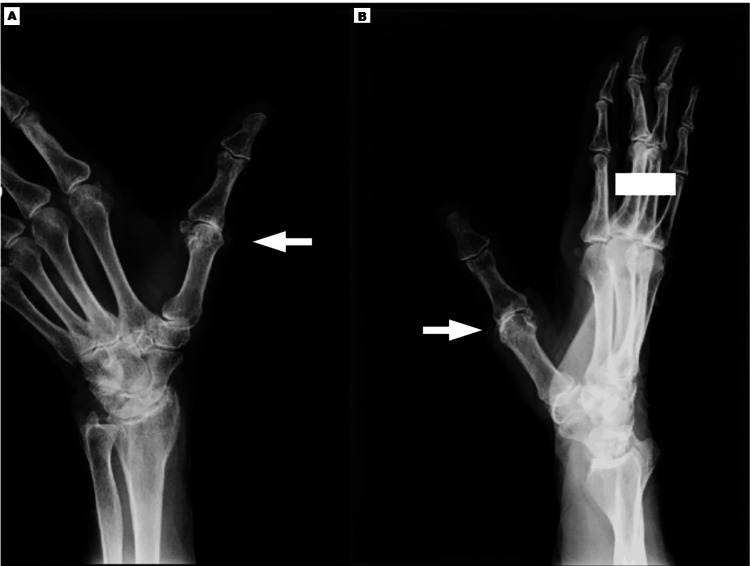
Radiographic Evaluation of First Metacarpophalangeal Joint Degeneration for Case 1 (A, B) Radiographic evaluation reveals advanced degeneration of the first metacarpophalangeal joint, consistent with Eaton-Littler stage III osteoarthritis.

An 84-year-old right-hand-dominant female patient presented with a nine-month history of debilitating right thumb pain, persistently rated 8-9/10 on the NRS [23-25]. Notably, the pain localized primarily to the anatomical snuffbox region with radiation along the first metacarpal, causing significant functional impairment (as evidenced by a QuickDASH questionnaire score [26] of 55). Radiographic evaluation demonstrated advanced first carpometacarpal joint degeneration consistent with Eaton-Littler stage III osteoarthritis (Figure 1). Conservative management had included three unsuccessful courses of physiotherapy focusing on joint mobilization and splinting, along with oral non-steroidal anti-inflammatory drugs (NSAIDs) (Celecoxib 200mg daily), which were discontinued due to intolerable epigastric pain and reflux symptoms.

**Figure 2:**
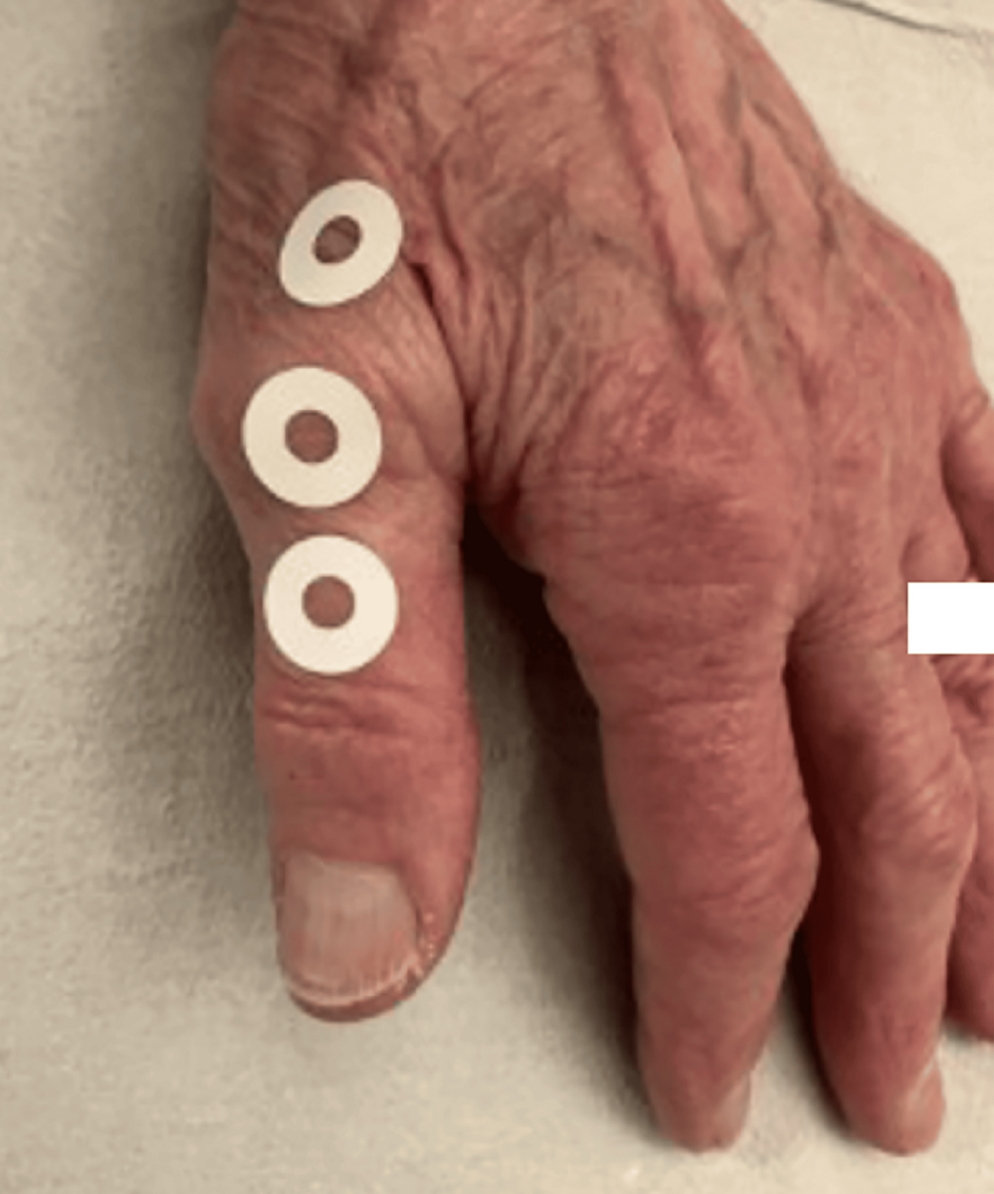
Landmark-Guided Fascial Injection Glucopuncture Sites for Chronic Thumb Pain (Case 1) The figure illustrates the sites of landmark-guided fascial injection with glucopuncture. Approximately 1 mL of 5% dextrose solution was injected into each of the three marked dots in this figure, targeting the subcutaneous tissue layers that include the superficial and deep fascia.

The patient underwent a series of palpation-guided subcutaneous injections of 5% dextrose in water (D5W). Using a 27-gauge 1/2-inch needle, 0.5 mL was administered per injection site targeting the superficial fascial layers surrounding the painful anatomical snuffbox region (Figure 2 ), with deliberate care taken to avoid intra-articular deposition. Injections were placed at 1-2 cm intervals along zones of maximal fascial thickening identified through systematic palpation. Treatment was administered weekly, with attention to fascial tension patterns and exact pain reproduction during physical examination.

**Figure 3:**
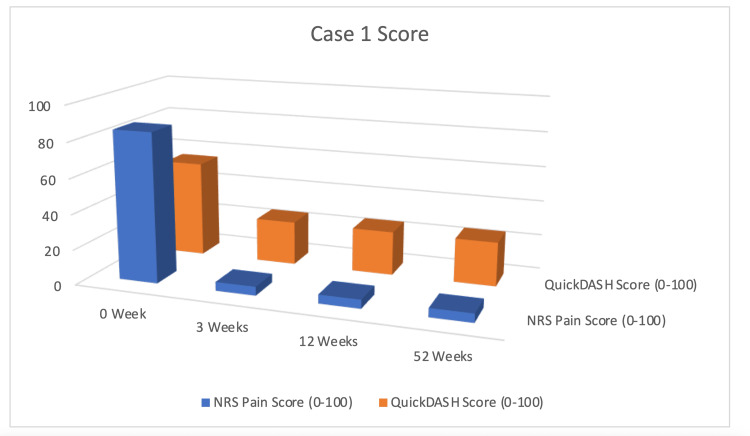
Clinical Progress Timeline for Case 1: Geriatric Basal Joint Osteoarthritis The graph depicts the progression of the Numerical Rating Scale (NRS) for pain (blue columns) and the Quick Disabilities of the Arm, Shoulder, and Hand (QuickDASH) score for function (orange columns) across three weekly glucopuncture sessions and the three-month and one-year follow-ups. A rapid, concurrent reduction in both pain and disability was observed, with outcomes exceeding the minimal clinically important difference (MCID) (15-point improvement for QuickDASH score) and being sustained at follow-up.

Reported clinical outcomes demonstrated progressive improvement: following two treatment sessions, the patient described approximately a 90% reduction in resting pain (NRS decreased from 8-9/10 to 0-1/10) and voluntarily discontinued all analgesic medications. By the third weekly session, functional capacity was subjectively improved dramatically with a QuickDASH score of 25-representing a 30-point reduction that numerically exceeds the established minimal clinically important difference (MCID) of 10-15 points for this measure. The three-month follow-up indicated sustained therapeutic benefit without the recurrence of baseline symptoms, though the absence of blinding or control limits causal attribution. The treatment efficacy was reportedly maintained at the one-year telephone follow-up (Figure 3).

Case 2: refractory myofascial pain syndrome in an elite combat athlete

**Figure 4:**
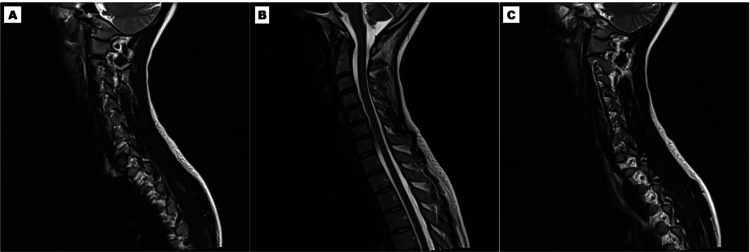
MRI Findings of Right C5-C6 Disc Herniation (Case 2) (A-C) Cervical MRI shows a right C5-C6 disc herniation without cord compression, offering only a partial explanation for the observed pain pattern.

A 34-year-old professional female kickboxer presented with a 12-month history of persistent right-sided cervicobrachial pain, consistently rated 8-9/10 on the NRS. Of clinical relevance, the pain distribution encompassed the posterior cervical triangle, superior medial scapular border, and distal triceps region. Functional impairment was severe, with a QuickDASH score of 56.8 and an NDI score of 77 [27-30]. The patient attributed symptom onset to repetitive rotational trauma during training activities. Initial management through multiple providers included cervical MRI demonstrating right C5-C6 disc herniation without cord compression, which provided only a partial explanation for the pain pattern (Figure 4). Unexpectedly, ultrasound-guided intradiscal and transforaminal epidural steroid injection with fluoroscopic confirmation (40 mg triamcinolone) at the affected level paradoxically exacerbated her symptoms, leading to refusal of further corticosteroid-based therapies.

Comprehensive evaluation revealed multiple objective findings: discrete myofascial trigger points in the right upper trapezius (1.5 cm depth), levator scapulae (2 cm depth), and long head of triceps (1 cm depth) that precisely reproduced her characteristic pain pattern upon digital pressure; measurable strength deficits (4/5 on manual muscle testing) in right shoulder abduction and elbow extension; and restricted active cervical rotation to 60° (normal > 80°) with positive pain provocation during shoulder cross-body adduction. These findings suggested a mixed pain generator profile combining cervical radiculopathy with secondary myofascial dysfunction.

**Figure 5:**
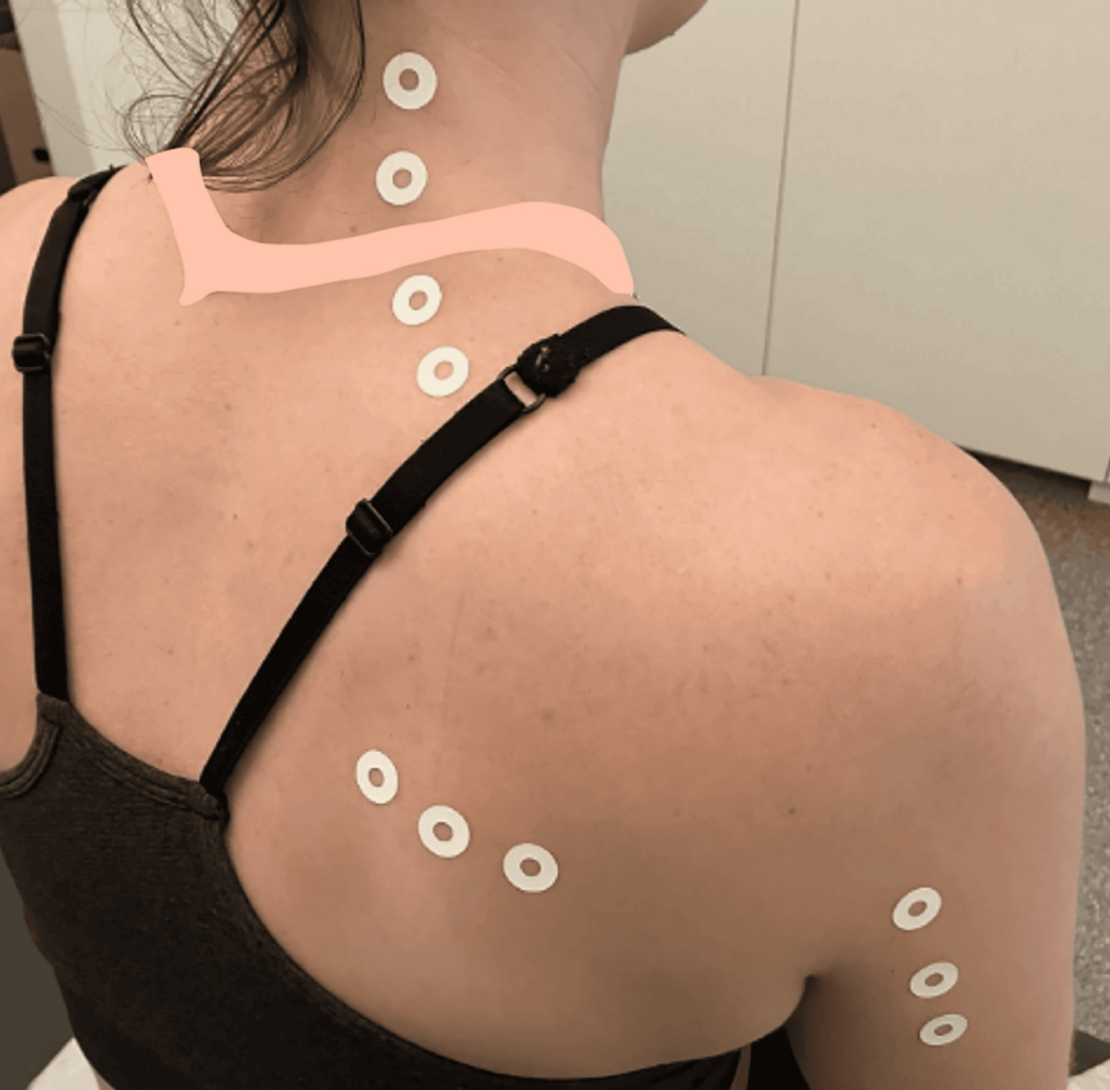
Injection Sites for Glucopuncture With 5% Dextrose for Chronic Neck, Shoulder, and Triceps Pain in an Athlete (Case 2) This figure illustrates the locations of the sore spots, which served as the injection sites for the glucopuncture with 5% dextrose solution in this athletic patient. Approximately 1 mL of 5% glucose solution was injected into each of these painful areas.

The patient underwent five weekly sessions of palpation-guided intramuscular 5% dextrose injections administered with a 27-gauge needle. Each trigger point received 1 mL of D5W delivered perpendicular to the skin surface, with injections spaced 1-2 cm apart throughout affected muscular regions (Figure 5). It should be noted that this was complemented by a structured rehabilitation program emphasizing scapulothoracic stabilization exercises and eccentric strengthening of the periscapular musculature, which may have contributed to outcomes.

Reported clinical outcomes demonstrated progressive improvement: by the second treatment session, the patient described 50% pain reduction (NRS 4/10) with QuickDASH improving to 31.8. Following the fourth session, she regained the ability to sleep on her right side. At treatment completion (fifth session), she achieved near-complete symptom resolution with pain at 0-1/10 on NRS, QuickDASH score of 11.3, and NDI of 33. The 45.5-point reduction in QuickDASH and 44-point improvement in NDI substantially exceed established MCID thresholds (10-15 points for QuickDASH; 10-13 points for NDI in radiculopathy). The patient successfully returned to full kickboxing training capacity without symptom recurrence at the six-week follow-up, with effects reportedly sustained at the one-year telephone follow-up.

Standardization of assessment and intervention protocols

A structured protocol guided all interventions. Injection sites were identified through systematic palpation of the affected regions, focusing on the detection of fascial thickening, ropiness, or taut bands characteristic of myofascial dysfunction. Each candidate site underwent quantitative validation via targeted pressure application, requiring exact reproduction of the patient's primary pain complaint (≥7/10 on NRS during palpation) before inclusion.

Technical execution differed according to tissue target: for fascial pain (Case 1), subcutaneous injections were administered at a shallow 15°-angle using 0.5 mL per site to maximize superficial distribution; for muscular trigger points (Case 2), perpendicular intramuscular delivery of 1 mL per site ensured adequate intramuscular deposition. All injections utilized 5% dextrose solution without additives, with sites spaced 1-2 cm apart to ensure comprehensive coverage of symptomatic territories.

**Figure 6:**
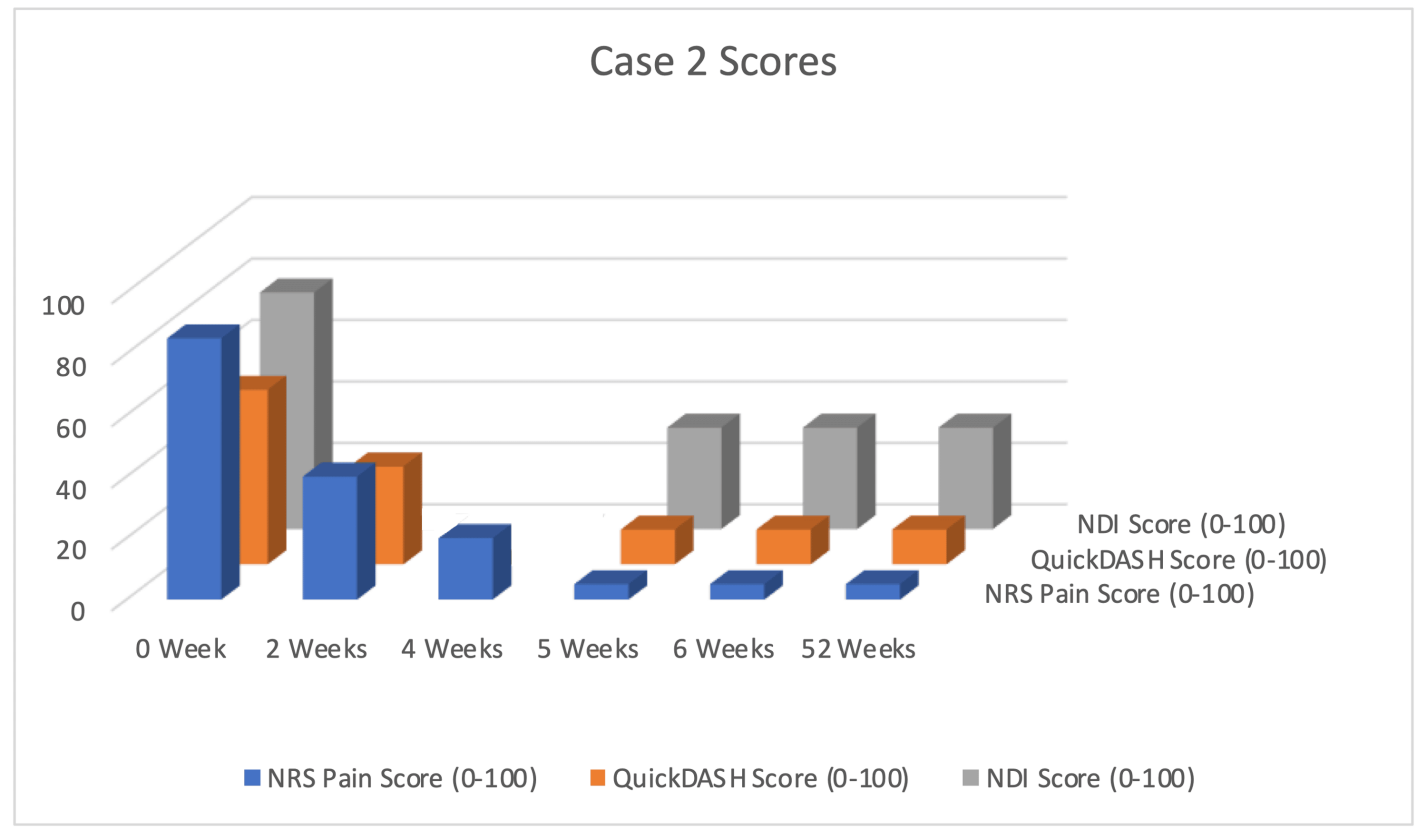
Clinical Progress Timeline for Case 2: Refractory Myofascial Pain in an Elite Athlete Timeline of pain and functional outcomes for Case 2. The graph shows the progression of the Numerical Rating Scale (NRS) for pain (blue columns), the Quick Disabilities of the Arm, Shoulder, and Hand (QuickDASH) score (orange columns), and the Neck Disability Index (NDI) score (gray columns) across five weekly treatment sessions and the six-week follow-up. The minimal clinically important difference (MCID) threshold for an improvement in QuickDASH is 15 points. Treatment consisted of glucopuncture injections combined with a structured rehabilitation program. A steady improvement in all metrics was observed, with functional scores demonstrating substantial, clinically significant improvement that was maintained at the one-year follow-up.

Follow-up assessments prioritized patient-reported functional metrics over structural imaging due to observed clinical-radiological discordance: in Case 1, the thumb pain distribution extended well beyond radiographic joint degeneration; in Case 2, cervical disc pathology failed to explain the diffuse triceps and periscapular pain pattern. Standardized evaluation included primary outcome assessment (≥50% NRS reduction at three months) and secondary functional metrics (QuickDASH, NDI) interpreted against established MCID thresholds. The observed improvements observed (Case 1: ΔQuickDASH 30; Case 2: ΔQuickDASH 45.5, ΔNDI 44) provide preliminary support for this fascial-muscular approach to complex pain syndromes where conventional structural diagnosis proves inadequate. Nevertheless, the lack of blinding and control groups necessitates cautious interpretation (Figure 6).

It is important to note that the improvements observed in this case occurred within a multimodal treatment context that included a structured rehabilitation program. Therefore, the specific contribution of glucopuncture cannot be isolated definitively. However, the rapid and substantial pain reduction (50% after the second session) preceding major functional gains, along with the direct targeting of her specific pain generators, suggests glucopuncture likely played a significant role in pain modulation, facilitating engagement in the rehabilitation process (Table 1).

**Table 1:** Clinical Summary of Two Cases Treated With Glucopuncture This table provides a comparative overview of patient demographics, clinical presentation, intervention details, and outcomes for the two presented cases. NRS: Numerical Rating Scale; QuickDASH: Quick Disabilities of the Arm, Shoulder, and Hand; NDI: Neck Disability Index; MCID: minimal clinically important difference; OA: osteoarthritis; NSAID: non-steroidal anti-inflammatory drugs.

Characteristic	Case 1: geriatric osteoarthritis	Case 2: elite athlete
Demographics	84-year-old, female, right-hand-dominant	34-year-old, female, professional kickboxer
Primary symptoms	Debilitating right thumb pain (NRS 8-9), radiation along the first metacarpal	Persistent right-sided cervicobrachial pain (NRS 8-9), involving neck, scapula, and triceps
Key findings	Eaton-Littler stage III OA on X-ray; failed physiotherapy & NSAIDs	C5-C6 disc herniation on MRI; myofascial trigger points; failed epidural injection
Intervention	Glucopuncture only: 3 weekly sessions of palpation-guided subcutaneous D5W injections around the anatomical snuffbox	Multimodal: 5 weekly sessions of intramuscular D5W trigger point injections + structured scapulothoracic rehabilitation
Outcomes	NRS: 8-9 → 0-1 (after 2 sessions); QuickDASH: 55 → 25 (Δ30, >MCID). Benefit sustained at 3-month and 1-year follow-ups	NRS: 8-9 → 0-1 (after 5 sessions); QuickDASH: 56.8 → 11.3 (Δ45.5, >MCID); NDI: 77 → 33 (Δ44, >MCID). Returned to full training at 6-week follow-up; benefit sustained at 1-year telephone follow-up

## Discussion

Glucopuncture emerges as a theoretically distinct therapeutic approach within the spectrum of minimally invasive musculoskeletal interventions, though comparative efficacy remains unproven. The procedure occupies a hypothetical middle ground between purely mechanical modalities like dry needling and biologically active therapies such as prolotherapy or platelet-rich plasma (PRP). When contrasted with perineural injection therapy (PIT), as pioneered by Dr. Lyftogt [31,32], potential differences in anatomical targeting and therapeutic intent become apparent. While PIT utilizes pH-buffered 5% dextrose administered subcutaneously along superficial nerve pathways [31,32], glucopuncture employs unbuffered dextrose to address both neural and non-neural structures, including deeper fascial layers and muscular trigger points. This broader targeting may prove particularly advantageous for complex pain syndromes where multiple tissue layers contribute to symptom generation, though this hypothesis requires validation.

The distinction between glucopuncture and traditional prolotherapy represents another area needing clarification. Prolotherapy, following the Hackett-Hemwall protocol [5,20,21,33-42], deliberately employs hyperosmolar solutions (typically 15%-25% dextrose) or irritants like phenol to provoke controlled tissue injury and subsequent healing. This stands in stark contrast to glucopuncture's use of iso-osmolar 5% dextrose, which is postulated to modulate pain through anti-inflammatory mechanisms rather than tissue destruction [20]. The clinical implications remain speculative: while prolotherapy aims primarily at structural reinforcement of ligaments and tendons, glucopuncture may offer more immediate analgesic effects through TRPV1 and Substance P downregulation [17,18,43-48].

When compared to dry needling, glucopuncture shares the mechanical benefits of needle disruption of myofascial trigger points but adds the theoretical pharmacological advantages of dextrose. Dry needling's reliance solely on mechanical effects could limit its efficacy in cases where neurogenic inflammation plays a prominent role [22], though direct comparisons are lacking. This proposed dual mechanism-combining mechanical disruption with neuromodulation-also presents a novel alternative to corticosteroid injections, which primarily suppress inflammation but carry risks of tissue atrophy and may not address underlying fascial dysfunction.

The contrast between glucopuncture and PRP therapy highlights unresolved questions in regenerative approaches. While both modalities aim to promote tissue healing, PRP utilizes the patient's own concentrated platelets-typically achieving 3-5 times baseline concentrations-to deliver a potent cocktail of growth factors including PDGF (platelet-derived growth factor), TGF-β (transforming growth factor beta), and VEGF (vascular endothelial growth factor) [49-52]. This biological richness theoretically enhances PRP's capacity to stimulate the healing cascade in neuromuscular damage through angiogenesis, fibroblast proliferation, and extracellular matrix remodeling [49,51,53]. In contrast, glucopuncture relies solely on the physicochemical properties of 5% dextrose (D5W), raising questions about its comparative regenerative potential.

The safety profile of glucopuncture appears favorable in this limited sample, with approximately 20% of patients experiencing a transient "reaction phase" of increased pain, typically resolving within 72 hours-a phenomenon less frequently reported with dry needling but more common than with PIT [54]. Crucially, this profile suggests a favorable risk-benefit ratio compared to common alternatives: unlike NSAIDs, it poses no risk of gastrointestinal or renal complications, and unlike corticosteroid injections, it avoids the potential for tissue weakening or atrophy [55]. Furthermore, glucopuncture requires no special preparation beyond basic sterile technique, enhancing its accessibility, though practitioners must note the lack of large-scale safety data.

This report has several limitations, inherent to its design as a case series. Most significantly, the open-label, uncontrolled nature means the observed improvements cannot be definitively attributed to the biochemical action of dextrose alone. The potent contributions of non-specific effects-including the placebo response, the therapeutic ritual of the injection procedure itself, and the natural history of the conditions-must be explicitly acknowledged as primary confounding factors. This is particularly relevant for Case 2, where glucopuncture was administered alongside a targeted rehabilitation program. While the temporal relationship between the injections and rapid pain reduction is suggestive of a specific effect, the synergistic contribution of exercise cannot be discounted. Future controlled studies with separate intervention arms are necessary to isolate the specific efficacy of glucopuncture.

The absence of standardized protocols for dextrose concentration, injection volume, and treatment frequency across studies complicates generalizability. Furthermore, the relative contributions of mechanical needle effects versus dextrose's pharmacological actions remain unquantified. After all, the two presented cases, while compelling, cannot establish efficacy or superiority over existing therapies.

Implications for future research and study design

The promising outcomes and technical protocol described in these cases provide a foundational framework for designing rigorous controlled trials to validate glucopuncture's efficacy. Future research should move beyond descriptive reports to address the specific confounders and questions raised here.

We propose the following specific study designs informed by our current findings. Key priorities include establishing efficacy through randomized controlled trials (RCTs), developing standardized protocols, and defining optimal patient selection criteria.

Placebo-Controlled Trials for Efficacy

To isolate the effect of dextrose from needle stimulation and placebo, a double-blind, RCT comparing palpation-guided glucopuncture to sham dry needling (using a blunted needle) or subcutaneous saline injections is essential. The rapid improvement seen in our cases, particularly in Case 1, suggests that such a trial could be conducted over a relatively short timeframe (e.g., 4-6 weeks) with NRS and functional scores like QuickDASH as primary outcomes.

Factorial Designs for Multimodal Therapy

For complex conditions like that presented in Case 2, a 2 x 2 factorial RCT design would be highly informative. This would randomize patients to one of four groups: (1) glucopuncture alone, (2) rehabilitation alone, (3) glucopuncture + rehabilitation, and (4) a control group. This design can statistically evaluate the individual contribution of each component and any synergistic interaction between them, directly addressing the confounding factor present in our report.

Mechanistic Studies

To validate the proposed neuropharmacological hypotheses, future studies should incorporate biomarker assays (e.g., Substance P and inflammatory cytokines) from microdialysates or serum samples pre- and post-intervention. Furthermore, ultrasound elastography could be used as an objective tool to quantify changes in fascial stiffness and mobility ("fascintegrity") following treatment.

Comparative Effectiveness Research

Once a specific effect is established, head-to-head trials against active comparators like dry needling or standard prolotherapy will be crucial to define glucopuncture's relative position in the therapeutic arsenal for musculoskeletal pain. By building upon the protocol standardization and outcome measures presented here, these targeted research strategies can translate our preliminary findings into robust evidence.

## Conclusions

The findings from these cases are hypothesis-generating, providing the preliminary clinical observations necessary to justify larger, controlled studies; they are not confirmatory evidence. Within this context, glucopuncture emerges as a clinically viable intervention for refractory musculoskeletal pain, with over 30 years of practical experience involving more than 100,000 patients. Its dual-action approach addresses both fascial dysfunction and neurogenic inflammation, effectively bridging a critical gap in managing complex pain syndromes where structural pathology and functional impairment coexist. Based on these preliminary findings, glucopuncture may fit within treatment algorithms as a potential option to consider after first-line conventional therapies (e.g., physiotherapy and oral analgesics) have failed, but before progressing to more invasive or costly interventions, particularly in resource-limited settings.

While preliminary, these findings align with extensive clinical observations regarding glucopuncture's safety, cost-effectiveness, and consistent outcomes across diverse populations. To transition glucopuncture from a promising experiential therapy to an evidence-based practice, future research must follow a structured pathway. This begins with mechanistic studies to validate its actions on neuropharmacological (e.g., TRPV1/Substance P) and biomechanical (e.g., fascintegrity) pathways. Subsequently, rigorous comparative efficacy trials against established modalities like dry needling or prolotherapy are essential. The development of reproducible, palpation-guided protocols will ensure global standardization, while health economic analyses will formally establish its value in resource-conscious healthcare systems. By achieving these goals, glucopuncture can be positioned as both a standalone treatment and a complementary approach within integrated pain management strategies, ensuring it reaches its full therapeutic potential based on robust scientific evidence.
